# HFM: A Hybrid Feature Model Based on Conditional Auto Encoders for Zero-Shot Learning

**DOI:** 10.3390/jimaging8060171

**Published:** 2022-06-16

**Authors:** Fadi Al Machot, Mohib Ullah, Habib Ullah

**Affiliations:** 1 Faculty of Science and Technology, Norwegian University of Life Science (NMBU), 1430 Ås, Norway; fadi.al.machot@nmbu.no (F.A.M.); habib.ullah@nmbu.no (H.U.); 2 Department of Computer Science, Norwegian University of Science and Technology, 2819 Gjøvik, Norway

**Keywords:** Zero-Shot Learning (ZSL), semantic space, conditional autoencoders, generative models, computer vision

## Abstract

Zero-Shot Learning (ZSL) is related to training machine learning models capable of classifying or predicting classes (labels) that are not involved in the training set (unseen classes). A well-known problem in Deep Learning (DL) is the requirement for large amount of training data. Zero-Shot learning is a straightforward approach that can be applied to overcome this problem. We propose a Hybrid Feature Model (HFM) based on conditional autoencoders for training a classical machine learning model on pseudo training data generated by two conditional autoencoders (given the semantic space as a condition): (a) the first autoencoder is trained with the visual space concatenated with the semantic space and (b) the second autoencoder is trained with the visual space as an input. Then, the decoders of both autoencoders are fed by the test data of the unseen classes to generate pseudo training data. To classify the unseen classes, the pseudo training data are combined to train a support vector machine. Tests on four different benchmark datasets show that the proposed method shows promising results compared to the current state-of-the-art when it comes to settings for both standard Zero-Shot Learning (ZSL) and Generalized Zero-Shot Learning (GZSL).

## 1. Introduction

Deep-learning-based models have brought tremendous advancement in different fields, including but not limited to computer vision [1,2], natural language processing [3], and satellite image processing [4]. In these research fields, deep-learning-based models achieved human-level capabilities. In fact, these developments are subject to higher quality and large-scale data. With the exponential growth of new classes in our real world, collecting large amounts of data driven by significant variations requires much cost. It is a key challenge to annotate sufficient training data for each class to exploit supervised learning [4,5]. Therefore, different learning paradigms with limited labeled data have been presented in the literature, namely semi-supervised learning [4], life-long learning [6], and active learning [7]. However, the capabilities of these paradigms are limited in exploring variations in the limited amount of labeled data. Generally, humans can recognize over 30,000 core item types [8] and many more sub-categories. Additionally, humans are also excellent at recognizing items without seeing any visual examples. This capability is the zero-shot learning problem in machine learning.

Zero-shot learning (ZSL) models [9,10,11] have recently emerged to identify unseen categories with no training data but with semantic descriptions of classes. The ZSL models can take into account situations when data are scarce [12,13]. In general, the ZSL models address this situation by learning either a visual-to-semantic mapping [14,15] or a semantic-to-visual mapping [16,17]. The general assumption is based on the observations that the visual space encodes the semantic space and that the semantic space encodes the visual space [15,18,19,20]. However, zero-shot learning is still a challenging research field since we need to predict unseen test categories that are never used when training the models [21,22,23]. For example, most ZSL methods like Deep Embedding Model (DEM) [24,25,26] discover direct embeddings from global features to the semantic space. However, the methods cannot capture the appearance relationships between different local regions in this way. The techniques could also ruin the diversity of visual modality due to highly overlapped semantic descriptions of various categories.

**Figure 1:**
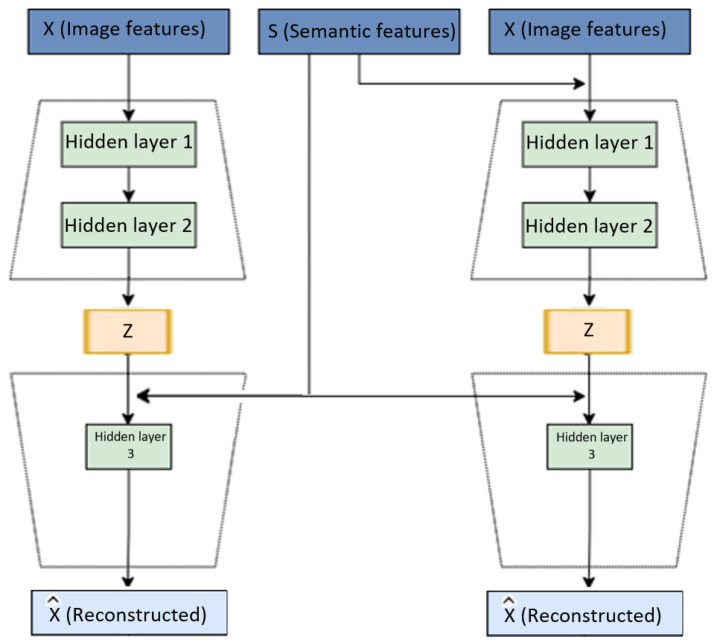
The proposed approach consists of two autoencoders. The first autoencoder is provided by the concatenated vectors of the visual and semantic spaces. The second autoencoder is provided by the visual features vectors only. Both autoencoders have a dense layer, followed by a dropout and a second dense layer. This is followed by another layer, which generates the values z. Activation functions are ReLU, and the activation functions for the last layer for both the encoder and the decoder are linear.

To cope with these challenges, we propose a Hybrid Feature Model (HFM) based on conditional autoencoders for zero-shot learning method to identify both seen and unseen classes via transferring knowledge from seen categories to unseen categories. Based on the observations [27] where a single conditional variational autoencoder is used, our method consists of two autoencoders that are depicted in Figure 1. The first autoencoder is provided by the concatenation of the visual and semantic spaces. The second autoencoder is provided by only the visual space. Our proposed method encodes the real data distribution efficiently. Therefore, our approach identifies the unbiased projection toward seen classes and produces close relationships between unseen samples and prototypes.

Most techniques fail to consider the discriminative information between the visual and semantic spaces. Thus, the significant insight is that our hybrid autoencoder approach may precisely represent the real data distribution of the query set in a fine-grained and dynamic manner. Especially, when the available samples are not driven by rich discriminative information. This can be exploited to enrich the diversity of data distribution and further improve the model accuracy. Furthermore, we explore both the visual and semantic spaces to encode diversified and discriminative modes of variation for learning a boosted classifier. Therefore, our method alleviates the problems when intra-class diversity and inter-class discriminability are lacking. Consequently, the proposed model presents promising results using a highly fine-grained dataset (see Section 5). In addition, the work shows that using multiple VAEs generate an improved discriminative image space where data are easier to separate for ZSL classification purposes.

The rest of the paper is divided into the following sections: in Section 2, we present the related works from the literature. In Section 3, we present our proposed method in detail. Experiments and experimental results on four benchmark datasets and a conclusion are presented in Section 4, Section 5, and Section 6, respectively.

## 2. Related Work

We classify the literature into two categories: embedding space-based zero-shot learning and feature generation-based zero-shot learning.

In the first category, Lampert et al. [12] presented attribute-based classification based on a high-level description that is phrased in terms of semantic attributes, such as the object’s color or shape. Norouzi et al. [13] introduced an image embedding system that mapped images into the semantic embedding space via a convex combination of the class label embedding vectors. However, the methods do not provide a natural mechanism for multiple semantic modalities to be fused and optimized jointly in an end-to-end structure. In [18], authors assumed that unseen categories come from unsupervised text corpora. Their method is based on the distributions of words in texts as a semantic space for understanding what objects look like. The method does not use the distribution information of samples. Therefore, the method cannot discover the cluster structure of samples. The authors [15] presented a visual-semantic embedding model trained to recognize visual objects using both labeled image data as well as semantic features gleaned from the unannotated text. They did not exploit the cluster relationship to rectify the biased sample-prototype relationship. Akata et al. [20] learned a function considering image and class embeddings. They used supervised attributes and unsupervised output embeddings either derived from hierarchies or learned from unlabeled text corpora. Xian et al. [21] introduced a latent embedding model for learning a compatibility function between image and class embeddings. Romera et al. [22] modeled the relationships between features, attributes, and classes as a two linear layers architecture, where the weights of the top layer are not learned but are given by the surrounding features. The researchers [23] embedded each class in the space of attribute vectors. Changpinyo et al. [28] aligned the semantic space to the model space that concerns itself with recognizing visual features. Kodirov et al. [29] presented a ZSL learning model based on a Semantic AutoEncoder (SAE). They projected a visual feature vector into the semantic space. The encoder and decoder may be linear and symmetric, which could not recognize or differentiate multiple features. Zhang et al. [24] used the visual space as the embedding space by considering the subsequent nearest neighbor search. The method [30] introduced an episode-based model for zero-shot learning. They trained their model within a set of episodes, each of which is modeled to simulate a zero-shot classification task. These methods have limited abilities to scale to large numbers of object categories. This limitation is partly due to the increasing complexity of collecting sufficient training data in the form of labeled images as the number of object categories grows.

In the second category, the methods learn to consolidate the visual samples for unseen classes. These methods first learn a conditional generative model considering, for example, Variational Autoencoder (VAE) and Generative Adversarial Networks (GAN). In addition, GAN-based approaches, e.g., f-VAEGAN-D2 [25] and TF-VAEGAN [26] show a competitive performance. In [25], authors proposed f-VAEGAN-D2, which combined VAEs and GANs to learn the marginal feature distribution of unlabeled images through an unconditional discriminator. However, the method cannot discover the class-based feature distribution from the available semantic information. In contrast to f-VAEGAN-D2 model, authors in [26] proposed the TF-VAEGAN model, which combined VAEs and GANs. However, they added a semantic embedding decoder to reconstruct the embedding space, which is used as a feedback module to improve the output of the Generator of the GAN. However, GANs and their derivatives show training instability, while VAE is more stable [31]. Mishra et al. [27] generated the samples from the given attributes, using a conditional variational autoencoder, and exploited the generated samples to classify the unseen classes.

Our proposed method falls into the feature generation-based zero-shot category driven by stability during training. The approach also encodes complex data distribution efficiently. It demonstrates that for specific test situations (see Section 5), a hybrid model consisting of two VAEs can outperform a GAN-VAE model with less training effort. Excluding the Kullback–Leibler (KL) divergence from the conditional VAE loss yields enhanced discriminative image features for classifying unseen classes in ZSL settings, which is promising. A limitation of the proposed approach is that the proposed model lacks a feedback module that can be coupled with the decoder to improve the reconstructed image space. To show the strength of our proposed method, we perform a comparison with a set of methods [12,13,15,18,20,21,22,23,24,25,26,27,28,29,30]. The reason for choosing these methods for comparison is three-fold. Firstly, they belong to both categories in the literature. Secondly, they represent different techniques. Lastly, these methods represent older and new techniques in the literature. We also compare our method with [19]. The considered approach is reinforcement learning for training image captioning methods. The comparison with this method would highlight the generalization capability of our approach.

## 3. A Hybrid Feature Model

### 3.1. Problem Definition

The basic idea of any ZSL approach is to build a model which maps information from the seen to unseen classes based on a semantic description of the unseen classes. In other word, zero-shot learning is needed when there are no labeled training examples for all classes under observation. Therefore, the available dataset is split into two groups, a training subset (seen classes) $Y_{seen}=\left\{y_{seen}^{1},y_{seen}^{2},\ldots ,y_{seen}^{n}\right\}$, and unseen classes $Y_{unseen}=\left\{y_{unseen}^{1},y_{unseen}^{2},\ldots ,y_{unseen}^{m}\right\}$ subset, where *n* refers to the number of seen classes and *m* refers to the number of unseen classes. In addition, the assumption $Y_{seen}\cap Y_{unseen}=\phi$ should hold. In such a situation, the task is to build a model $R^{d}\to Y_{unseen}$ using only the training subset and able to classify the unseen classes. Afterward, the trained classifier should be applied on test data of unseen classes under the zero-shot settings $Y_{seen}\cap Y_{unseen}=\phi$. Consequently, zero-shot learning provides a new technique to overcome obstacles, such as the lack of training examples aiming at increasing a learning system’s capability to deal with unexpected events in the same way that people do.

Most state-of-the-art techniques solve the ZS problem by embedding the training data feature space and the semantic representation of class labels in some vector space to preserve the similarity. Then, unseen classes can be classified as nearest-neighbor search problems. In the generalized zero-shot case, we seek to design a more generic model $R^{d}\to Y_{seen}\cup Y_{unseen}$, that is able to categorize or classify the seen and unseen classes appropriately.

### 3.2. Approach

The Variational Autoencoder [32] consists of a decoder and an encoder. The encoder and the decoder are trained to aim at maximizing a goal which is known as the Evidence Lower Bound (ELBo). In both the encoder and the decoder, the variable *z* represents the hidden, latent space and the variable *x* represents the data. In addition, the encoder $q_{\Phi }\left(z|x\right)$ consists of parameters Φ and maps from data space to latent space and a decoder $p_{\theta }\left(x|z\right)$ which consists of the parameters θ and maps from latent space to data space. The lower bound for p(x) can be written as:

(1) $$L\left(\Phi ,\theta ;x\right)=-KL(q_{\Phi }\left(z|x\right)||p_{\theta }\left(z\right))+E_{q\Phi (z|x)}\left[logp_{\theta }\left(x|z\right)\right]$$

In Equation (1), *KL* denotes the Kullback–Leibler divergence between the encoder’s distribution $q_{\Phi }\left(z|x\right)$ and $p_{\theta }\left(z\right)$.

Conditional Variational Autoencoders (CVAE) [33] consists of the encoder and the decoder that can be conditioned to additional variables like the variable *x* (data) and the condition variable *c*. Thus, it is possible to generate samples following desired properties that might be encoded by *c* also. The loss function can be given as:

(2) $$L\left(\Phi ,\theta ;x,c\right)=-KL(q_{\Phi }\left(z|x,c\right)||p_{\theta }\left(z|c\right))+E_{q\Phi (z|c)}\left[logp_{\theta }\left(x|z,c\right)\right]$$

In this work, our loss function considers only the reconstruction term which is the Mean Squared Error (MSE).

We chose to use such a loss function because researchers in [34,35,36], showed that the *KL* divergence in the standard conditional variational autoencoder (see Equation (1)) does not allow the model to use the latent variables in many situations effectively. In this paper, we show that dropping the Kullback–Leibler (KL) term from the Variational Autoencoder [32] shows promising performance.

Algorithm 1 shows the training steps. Firstly, the algorithm requires the image features $X_{seen}$, the labels of the image features (visual space) $Y_{seen}$, and the vectors of the semantic space $S_{seen}$. Then the first autoencoder $Autoencoder_{1}$ is trained using $X_{seen}$ combined with $S_{seen}$ and learns the latent space *z* to generate $\hat{x}$ given $S_{seen}$. Then the second autoencoder $Autoencoder_{2}$ is trained using the $X_{seen}$ and learns the latent space *z* to generate ${\hat{X}}_{seen}$ given $S_{seen}$.
**Algorithm 1** Training**Require:** $X_{seen},Y_{seen},S_{seen}$**Ensure:** $Autoencoder_{1},Autoencoder_{2}$ Train the conditional model ($Autoencoder_{1},condition\;\;is\;\;S_{seen}$)   ($X_{seen},S_{seen}\to X_{seen}$) Train the conditional model ($Autoencoder_{2},condition\;\;is\;\;S_{seen}$)   ($X_{seen}\to \;\;X_{seen}$)

Algorithm 2 shows the detailed steps to classify the unseen classes. The algorithm requires the first autoencoder $Autoencoder_{1}$, the second autoencoder $Autoencoder_{2}$, and the semantic vectors of unseen labels $S_{unseen}$. Then, the encoder of the first autoencoder $Autoencoder_{1}$ will estimate $q(z^{\left(i\right)}|x^{\left(i\right)},S_{Y_{i}})$ but the input of the encoder is the image feature concatenated with the semantic vectors. Then, the decoder of $Autoencoder_{1}$ tries to reconstruct *x* using a sampled z from a standard normal distribution concatenated with $S_{unseen}$. Then, the encoder of the second autoencoder $Autoencoder_{2}$ will estimate $q(z^{\left(i\right)}|x^{\left(i\right)},S_{Y_{i}})$ but the input of the encoder is only the image feature space. Then, the decoder of $Autoencoder_{2}$ tries to reconstruct *x* using a sampled z from a standard normal distribution concatenated with $S_{unseen}$. The generated $\hat{x}$ from both autoencoders will be concatenated to form the pseudo training data for a support vector machine. Then, the Support Vector Machine (SVM) is trained, and its parameters are fitted. We use it to predict the performance using the unseen test classes.
**Algorithm 2** Unseen classes classification**Require:** $Autoencoder_{1},Autoencoder_{2},X_{unseen},S_{unseen}$, $Y_{unseen}$**Ensure:** classLabel $TrainingSet_{Autoenc_{1}}=\Phi$  **for** $y_{unseen}\in Y_{unseen}$ **do**   **for** iinNumOfSamples **do**   #samplefromaGaussiandistribution   z ~N(0,1)   #Concanetatezandtheunseensemanticclasslabel   $tmpV_{i}=S_{unseen}\circ z$   #Generateapseudo−samplefromthefirstautoencoder   $PseudoX_{i}\leftarrow Decoder_{Autoencoder_{1}}\left(tmpV_{i}\right)$   $\#\;\;Add\;\;the\;\;sample\;\;and\;\;the\;\;unseen\;\;class\;\;label\;\;to\;\;TrainingSet_{Autoenc_{1}}$   $TrainingSet_{Autoenc_{1}}\leftarrow TrainingSet_{Autoenc_{1}}\cup \left(PseudoX_{i},y_{unseen}\right)$  **end for** **end for** $TrainingSet_{Autoenc_{2}}=\Phi$ **for** $y_{unseen}\in Y_{unseen}$ **do**  **for** iinNumOfSamples **do**   #samplefromaGaussiandistribution   z ~N(0,1)   #Concanetatezandtheunseensemanticclasslabel   $tmpV_{i}=S_{unseen}\circ z$   #Generateapseudo−samplefromthesecondautoencoder   $PseudoX_{i}\leftarrow Decoder_{Autoencoder_{2}}\left(tmpV_{i}\right)$   $\#\;\;Add\;\;the\;\;sample\;\;and\;\;the\;\;unseen\;\;class\;\;label\;\;to\;\;TrainingSet_{Autoenc_{2}}$   $TrainingSet_{Autoenc_{2}}\leftarrow TrainingSet_{Autoenc_{2}}\cup \left(PseudoX_{i},y_{unseen}\right)$  **end for** **end for** $S_{training}=TrainingSet_{Autoenc_{1}}\cup TrainingSet_{Autoenc_{2}}$ fit SVM model using $S_{training}$ Use the trained SVM model classLabel = SVM($X_{unseen}$)

## 4. Experiments

In the field of ZSL, there are well-known benchmark datasets. Therefore, we selected four of them to test the performance of the proposed approach. We used, SUN Attribute (SUN) dataset [37] which consists of 14340 images, 645 classes are seen and 72 unseen. Caltech-UCSD-Birds (CUB) [38] which consists of 11788 images, 150 classes are seen and 50 unseen. In addition, we used Animals with Attributes1 and Animals with Attributes2 (AwA-1) and (AwA-2) [39] datasets. AwA-1 consists of 30475 images, 40 classes are seen and 10 unseen. AwA-2 dataset consists of 37322 images, 40 classes are seen and 10 unseen.

**Figure 2:**
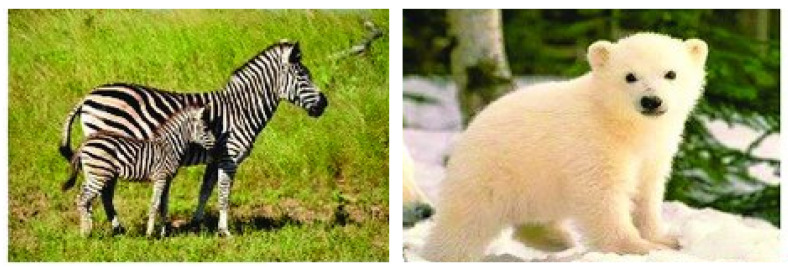
Examples from Animals with Attributes (AWA) dataset.

**Figure 3:**
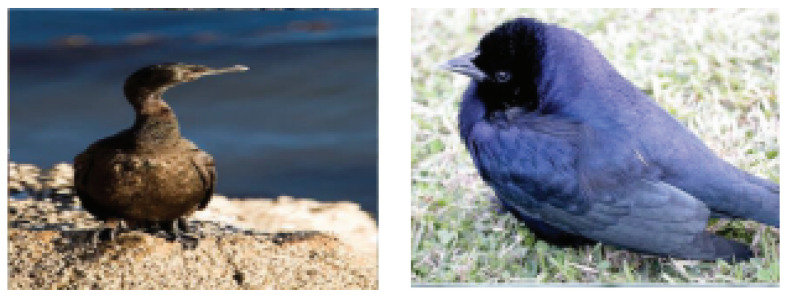
Examples from Caltech-UCSD-Birds (CUB) dataset.

**Figure 4:**
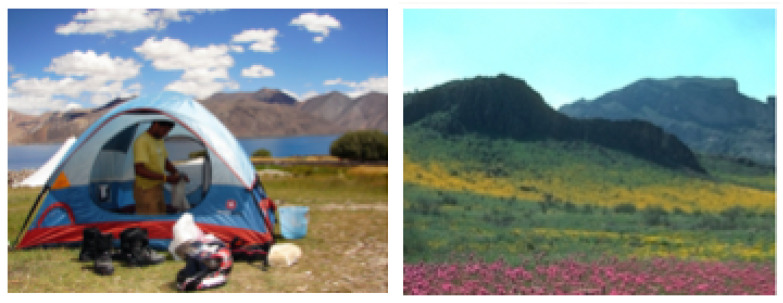
Examples from SUN Attribute (SUN) dataset.

Figure 2, Figure 3 and Figure 4 show examples from AwA, CUB and SUN datasets, respectively.

Regarding the visual space, we explored the Residual Neural Network 101 (ResNet101) features [39]. Concerning the semantic space, we rely on the semantic space vectors given by the authors of those datasets. Both autoencoders have a dense layer, followed by a dropout and a second dense layer. This is followed by another layer, which generates the values z. Activation functions are ReLU, and the activation functions for the last layer for both the encoder and the decoder are linear. In addition, we use the keras [40] framework in combination with the tensorflow backend [41] for implementation.

In our model, hyper-parameters are divided into two categories. The network hyper-parameters and the Support Vector Machine (SVM) cost parameter. The network hyperparameters are set to batch size equal to 50, the size of the latent variable is 50, and the optimizer is Adam [42]. The number of generated samples for each class is equal to 200. Cross-validation on training classes is used to determine the latent variable size. The SVM cost parameter is set to 100. To calculate the overall accuracy, we used the per-class average:

(3) $$acc_{average}^{per-class}=\frac{1}{\left|Y\right|}\Sigma_{i=0}^{\left|Y\right|}\left(\frac{N_{correct_{class}}^{class_{i}}}{N_{Total}^{class_{i}}}\right)$$

Regarding the GZSL, we explored the generalized zero-shot situation [43]. We kept aside 20% of the data from the training images and trained the model using the remaining 80% of the data. The SVM is trained using both the seen and the unseen classes to avoid biased performance toward seen classes. For Generalized Zero-Shot Learning (GZLS), we followed the recommendation in [44] to consider the harmonic mean of the accuracy between seen and unseen classes.

## 5. Results and Discussion

Table 1 shows the state-of-the-art comparison on four datasets using per-class average and the suggested splits from [39]. Our HFM model shows classification scores of 69.5%, 65.0%, 65.5%, and 53.8% on CUB, AwA1, AwA2, and SUN, respectively. For the ZSL settings, Table 1 shows that f-VAEGAN-D2 [25] and TF-VAEGAN [26] performed the best for AwA2 and SUN datasets. However, our model outperforms them using the CUB dataset. This result is promising because our model showed an improved performance using the highly fine-grained CUB dataset, which means that the generated pseudo-images gave separable output space. We attribute this to excluding Kullback Leibler divergence and to the hybrid nature of our reconstructed image feature space. Unfortunately, the authors of f-VAEGAN-D2 and Tf-VAEGA did not provide any results related to AwA1 dataset.

**Figure 5:**
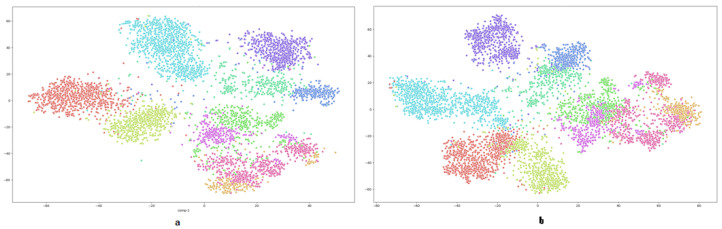
This figure visualizes the image feature space of the AwA-1 dataset (each color denotes a label), (**a**) shows t-SNE real test data visualization and (**b**) shows the test data generated from the proposed approach.

As shown in Figure 5, we visually inspect the image feature vectors produced by our model for each class using the t-SNE [45] technique, and we compare them to the original test image feature vectors for the AwA-1 dataset. As a result, we could observe that the proposed approach can accurately simulate the underlying images. In addition, we could observe that the reconstructed image features did not exclude many modes compared to the real distribution.

Table 2 shows the result of the Generalized Zero-Shot Learning (GZSL) compared to the well-known state-of-the-art approaches. The table shows comparable performance for the CUB and AwA2 dataset. However, the proposed approach showed better performance using AwA1 dataset. Table 2 shows that our HFM model has a harmonic mean score of 43.4%, 61.6%, 63.4%, and 29.7% on CUB, AwA1, AwA2, and SUN, respectively. The results of the Generalized Zero-Shot learning can be explained because of using ELBo without KL divergence (KL-free) is still theoretically a valid target for generative modeling using VAEs [35].

Table 3 shows the results for every autoencoder on four datasets under the ZSL setting. The results of the table confirm that combining the image feature spaces that are generated using both autoencoders improved the overall performance significantly.

Table 4 shows the results of the Generalized zero-shot setting (GZSL) that are calculated based on per-class average using seen classes, unseen classes, and harmonic mean.

Furthermore, other recent works, e.g., AFRNet [46] and GEM-ZSL [47] showed competitive results compared to our approach using different experimental settings. In AFRNet [46], authors proposed an adversarial network consisting of a residual generator, a prototype predictor, and a discriminator to synthesize compact semantic visual features for ZSL. Furthermore, authors in GEM-ZSL [47], their goal is the estimation of the real human gaze position to determine the visual attention areas for recognizing an unseen object using the semantic description of attributes. Thus, a feedback module combined with the decoder of each VAE may improve the overall performance of the GZSL problem.

## 6. Conclusions

Zero-Shot learning is related to building machine learning models that can classify or predict classes (labels) that are not included in the training set. In this work, a generative zero-shot learning model is developed. The model can be extended to different use case scenarios. In addition, this work provided intensive tests and detailed coverage of state-of-the-art technology. According to our results, the model shows promising results in some cases compared to the state-of-the-art methods considering three benchmark datasets, even in the case of generalized zero-shot learning. Our proposed method showed that: (a) excluding the Kullback–Leibler (KL) divergence from the conditional VAE loss synthesizes discriminative image features for classifying unseen classes in ZSL problem settings, (b) Using multiple VAEs generates an improved discriminative image space where data are easier to separate for classification purposes. Moreover, a limitation of the proposed approach is that the proposed model lacks a feedback module that can improve the reconstructed pseudo-image space. In our future work, we will add a feedback module and extend our generative model to combine the generative model with an additional embedding model. It means the model maps both the real and the pseudo-generated samples produced by the generative model into a new embedding space where classes are better separable.

## Figures and Tables

**Table 1:** State-of-the-art comparison on four datasets using the per-class average under the ZSL setting.

Model	CUB	AwA1	AwA2	SUN
DAP [12]	40.0	44.1	46.1	39.9
IAP [12]	24.0	35.9	35.9	19.4
ConSE [13]	34.3	45.6	44.5	38.8
CMT [18]	34.6	39.5	37.9	39.9
SSE [19]	43.9	60.1	61.0	51.5
DeViSE [15]	52.0	54.2	59.7	56.5
SJE [20]	53.9	65.6	61.9	53.7
LATEM [21]	49.3	55.1	55.8	55.3
ESZSL [22]	53.9	58.2	58.6	54.5
ALE [23]	54.9	59.9	62.5	58.1
SYNC [28]	55.6	54.0	46.6	56.3
SAE [29]	33.3	53.0	54.1	40.3
Relation Net [30]	55.6	68.2	64.2	-
DEM [24]	51.7	68.4	67.1	61.9
f-VAEGAN-D2 [25]	61.0	—	71.1	64.7
TF-VAEGAN [26]	64.9	—	72.2	66.0
CVAE [27]	52.1	71.4	65.8	61.7
HFM (Ours)	69.5	65.0	65.5	53.8

**Table 2:** Results of Generalized Zero-Shot Learning (GZSL) settings.We used the harmonic mean of accuracy on both seen and unseen classes as a measure.

Model	CUB	AwA1	AwA2	SUN
DAP [12]	3.3	0.0	0.0	7.2
IAP [12]	0.4	4.1	1.8	1.8
ConSE [13]	3.1	0.8	1.0	11.6
CMT [18]	8.7	15.3	15.9	13.3
SSE [19]	14.4	12.9	14.8	4.0
DeViSE [15]	32.8	22.4	27.8	20.9
SJE [20]	33.6	19.6	14.4	19.8
LATEM [21]	24.0	13.3	20.0	19.5
ESZSL [22]	21.0	12.1	11.0	15.8
ALE [23]	34.4	27.5	23.9	26.3
SYNC [28]	19.8	16.2	18.0	13.4
SAE [29]	13.6	3.5	2.2	11.8
Relation Net [30]	47.0	46.7	45.3	—
DEM [24]	29.2	47.3	45.1	25.6
f-VAEGAN-D2 [25]	53.6	—	63.5	41.3
TF-VAEGAN [26]	58.1	—	66.6	43.0
CVAE [27]	34.5	47.2	51.2	26.7
HFM (Ours)	43.4	61.6	63.4	29.7

**Table 3:** Results for each autoencoder on four datasets under the ZSL setting. The performance is evaluated using the per-class average.

Dataset	Autoencoder_1_	Autoencoder_2_	Both
AWA1	63.6	60.0	65.0
AWA2	58.6	58.4	65.5
CUB	68.5	58.9	69.5
SUN	50.6	51.4	53.8

**Table 4:** Results of Generalized Zero-Shot setting (GZSL) that are calculated based on per-class average using seen classes, unseen classes, and harmonic mean.

Dataset	Seen	Unseen	Harmonic Mean
AWA1	75.7	52.0	61.6
AWA2	80.9	49.7	63.4
CUB	57.9	34.7	43.4
SUN	75.3	18.5	29.7

## References

[1] Yan T., Li H., Sun B., Wang Z., Luo Z. Discriminative Feature Mining and Enhancement Network for Low-resolution Fine-grained Image Recognition. IEEE Trans. Circuits Syst. Video Technol..

[2] Shagdar Z., Ullah M., Ullah H., Cheikh F.A. Geometric Deep Learning for Multi-Object Tracking: A Brief Review. Proceedings of the 2021 9th European Workshop on Visual Information Processing (EUVIP).

[3] Wu C., Li X., Guo Y., Wang J., Ren Z., Wang M., Yang Z. (2022). Natural language processing for smart construction: Current status and future directions. Autom. Constr..

[4] Ullah H., Ahmed T.U., Ullah M., Cheikh F.A. (2021). IR-SSL: Improved Regularization Based Semi-Supervised Learning For Land Cover Classification. Proceedings of the 2021 IEEE International Conference on Image Processing (ICIP).

[5] Aljaloud A.S., Ullah H. (2021). IA-SSLM: Irregularity-Aware Semi-Supervised Deep Learning Model for Analyzing Unusual Events in Crowds. IEEE Access.

[6] Zhao T., Wang Z., Masoomi A., Dy J. (2022). Deep Bayesian Unsupervised Lifelong Learning. Neural Netw..

[7] Hunter R.A., Pompano R.R., Tuchler M.F. (2022). Alternative Assessment of Active Learning. Active Learning in the Analytical Chemistry Curriculum.

[8] Biederman I. (1987). Recognition-by-components: A theory of human image understanding. Psychol. Rev..

[9] Min S., Yao H., Xie H., Wang C., Zha Z.J., Zhang Y. Domain-aware visual bias eliminating for generalized zero-shot learning. Proceedings of the IEEE/CVF Conference on Computer Vision and Pattern Recognition.

[10] Han Z., Fu Z., Chen S., Yang J. Contrastive embedding for generalized zero-shot learning. Proceedings of the IEEE/CVF Conference on Computer Vision and Pattern Recognition.

[11] Zhang J., Li Q., Geng Y.A., Wang W., Sun W., Shi C., Ding Z. (2022). A zero-shot learning framework via cluster-prototype matching. Pattern Recognit..

[12] Lampert C.H., Nickisch H., Harmeling S. (2013). Attribute-based classification for zero-shot visual object categorization. IEEE Trans. Pattern Anal. Mach. Intell..

[13] Norouzi M., Mikolov T., Bengio S., Singer Y., Shlens J., Frome A., Corrado G.S., Dean J. (2013). Zero-shot learning by convex combination of semantic embeddings. arXiv.

[14] Gao R., Hou X., Qin J., Shen Y., Long Y., Liu L., Zhang Z., Shao L. Visual-Semantic Aligned Bidirectional Network for Zero-Shot Learning. IEEE Trans. Multimed..

[15] Frome A., Corrado G.S., Shlens J., Bengio S., Dean J., Ranzato M., Mikolov T. (2013). Devise: A deep visual-semantic embedding model. Adv. Neural Inf. Process. Syst..

[16] Annadani Y., Biswas S. Preserving semantic relations for zero-shot learning. Proceedings of the IEEE Conference on Computer Vision and Pattern Recognition.

[17] Vyas M.R., Venkateswara H., Panchanathan S. (2020). Leveraging Seen and Unseen Semantic Relationships for Generative Zero-Shot Learning. Proceedings of the European Conference on Computer Vision.

[18] Socher R., Ganjoo M., Manning C.D., Ng A. (2013). Zero-shot learning through cross-modal transfer. Adv. Neural Inf. Process. Syst..

[19] Zhang L., Sung F., Liu F., Xiang T., Gong S., Yang Y., Hospedales T.M. (2017). Actor-critic sequence training for image captioning. arXiv.

[20] Akata Z., Reed S., Walter D., Lee H., Schiele B. Evaluation of output embeddings for fine-grained image classification. Proceedings of the IEEE Conference on Computer Vision and Pattern Recognition.

[21] Xian Y., Akata Z., Sharma G., Nguyen Q., Hein M., Schiele B. Latent embeddings for zero-shot classification. Proceedings of the IEEE Conference on Computer Vision and Pattern Recognition.

[22] Romera-Paredes B., Torr P. (2015). An embarrassingly simple approach to zero-shot learning. Proceedings of the International Conference on Machine Learning.

[23] Akata Z., Perronnin F., Harchaoui Z., Schmid C. (2015). Label-embedding for image classification. IEEE Trans. Pattern Anal. Mach. Intell..

[24] Zhang L., Xiang T., Gong S. Learning a deep embedding model for zero-shot learning. Proceedings of the IEEE Conference on Computer Vision and Pattern Recognition.

[25] Xian Y., Sharma S., Schiele B., Akata Z. f-vaegan-d2: A feature generating framework for any-shot learning. Proceedings of the IEEE/CVF Conference on Computer Vision and Pattern Recognition.

[26] Narayan S., Gupta A., Khan F.S., Snoek C.G., Shao L. (2020). Latent embedding feedback and discriminative features for zero-shot classification. Proceedings of the European Conference on Computer Vision.

[27] Mishra A., Krishna Reddy S., Mittal A., Murthy H.A. A generative model for zero shot learning using conditional variational autoencoders. Proceedings of the IEEE Conference on Computer Vision and Pattern Recognition Workshops.

[28] Changpinyo S., Chao W.L., Gong B., Sha F. Synthesized classifiers for zero-shot learning. Proceedings of the IEEE Conference on Computer Vision and Pattern Recognition.

[29] Kodirov E., Xiang T., Gong S. Semantic autoencoder for zero-shot learning. Proceedings of the IEEE Conference on Computer Vision and Pattern Recognition.

[30] Sung F., Yang Y., Zhang L., Xiang T., Torr P.H., Hospedales T.M. Learning to compare: Relation network for few-shot learning. Proceedings of the IEEE Conference on Computer Vision and Pattern Recognition.

[31] Zhang T., Yang Z., Li D. (2022). Stochastic simulation of deltas based on a concurrent multi-stage VAE-GAN model. J. Hydrol..

[32] Kingma D.P., Welling M. (2013). Auto-encoding variational bayes. arXiv.

[33] Sohn K., Lee H., Yan X. (2015). Learning structured output representation using deep conditional generative models. Adv. Neural Inf. Process. Syst..

[34] Bowman S.R., Vilnis L., Vinyals O., Dai A.M., Jozefowicz R., Bengio S. (2015). Generating sentences from a continuous space. arXiv.

[35] Zhao S., Song J., Ermon S. (2017). Towards deeper understanding of variational autoencoding models. arXiv.

[36] Chen R.T., Li X., Grosse R.B., Duvenaud D.K. (2018). Isolating sources of disentanglement in variational autoencoders. Adv. Neural Inf. Process. Syst..

[37] Patterson G., Hays J. (2012). Sun attribute database: Discovering, annotating, and recognizing scene attributes. Proceedings of the 2012 IEEE Conference on Computer Vision and Pattern Recognition.

[38] Wah C., Branson S., Welinder P., Perona P., Belongie S. (2011). The Caltech-Ucsd Birds-200-2011 Dataset.

[39] Xian Y., Schiele B., Akata Z. Zero-shot learning-the good, the bad and the ugly. Proceedings of the IEEE Conference on Computer Vision and Pattern Recognition.

[40] Bursztein E., Chollet F., Jin H., Watson M., Zhu Q.S. Keras: The Python Deep Learning API. https://keras.io.

[41] Abadi M., Agarwal A., Barham P., Brevdo E., Chen Z., Citro C., Corrado G.S., Davis A., Dean J., Devin M. (2015). TensorFlow: Large-Scale Machine Learning on Heterogeneous Systems. tensorflow.org.

[42] Kingma D.P., Ba J. (2014). Adam: A method for stochastic optimization. arXiv.

[43] Chao W.L., Changpinyo S., Gong B., Sha F. (2016). An empirical study and analysis of generalized zero-shot learning for object recognition in the wild. Proceedings of the European Conference on Computer Vision.

[44] Xian Y., Lorenz T., Schiele B., Akata Z. Feature generating networks for zero-shot learning. Proceedings of the IEEE Conference on Computer Vision and Pattern Recognition.

[45] Van der Maaten L., Hinton G. (2008). Visualizing data using t-SNE. J. Mach. Learn. Res..

[46] Liu B., Dong Q., Hu Z. Zero-shot learning from adversarial feature residual to compact visual feature. Proceedings of the AAAI Conference on Artificial Intelligence.

[47] Liu Y., Zhou L., Bai X., Huang Y., Gu L., Zhou J., Harada T. Goal-oriented gaze estimation for zero-shot learning. Proceedings of the IEEE/CVF Conference on Computer Vision and Pattern Recognition.

